# Supplementary material for: No sex differences in adult telomere length across vertebrates: a meta-analysis
Source: R Soc Open Sci. 2020 Nov 11;7(11):200548. doi: 10.1098/rsos.200548 (PMC7735339; doi:10.1098/rsos.200548)
Supplement: Supplementary methods, results and references used in the meta-analyses [file rsos200548supp2.docx]

**Supplementary Method:**

Moderators used in the analysis:

To assess which factors could explain the direction and the magnitude of sex differences in TL across vertebrates, we included the following moderators as covariates or fixed factors for each meta-analysis:

- The *class* (corresponding to the taxonomic class of each species) was included in the model as a four-level factor “Bird”, “Mammal”, “Reptile” and “Fish” (that includes ray-finned fish and cartilaginous fish).
- The *method* of telomere measurement was included as a three-level factor: qPCR, TRF_in gel_ and TRF_southern blot_. The influence of the method of measurement was tested because it might be a source of heterogeneity among studies [1]. Indeed, the TRF (for Telomere Restriction Fragment) is an absolute measure of the mean TL in a sample of cells. This method uses restriction enzymes that do not cut within the telomere sequence to digest genomic DNA. Moreover, two different types of TRF exist: the TRF with in-gel hybridization (TRF_in gel_ here) and the TRF followed by a southern blot (TRF_southern blot_ here). In the TRF_in gel_ the probe binds to the single-stranded overhang of the telomere and therefore only telomeres are measured. Whereas in the TRF_southern blot_, because the DNA is denatured, the probe binds to telomere and also to interstitial telomeric sequences, which are telomeric repeats located within chromosomes. The qPCR, provides a relative measure of TL. Telomeric primers are used to amplify telomeric sequences and the amount of telomere sequence is then compared to the amount of a non-telomeric reference. As for the TRF_southern blot_, the qPCR amplifies both telomeres and interstitial telomeric sequences (See [2] for review).
- The *biological tissue* used for telomere measurement was included as a three-level factor: “Red blood Cell” (RBC) “White blood cell” (WBC) and a third category that included all other tissues coded as “Other tissue” because many tissues were included only once in the dataset (*e.g.* brain, spleen, muscle). The tissue was included in the model because TL can differ across biological tissues within a given individual [2], possibly in a sex-specific way. Note that RBC have not been sampled in mammals because red blood cells are not nucleated in this class of vertebrates.
- The type of *population* was included as a two-level factor (“Wild” *vs.* “Captive”). The response of individuals to captive conditions vary between sexes and among species [3], which might potentially be associated with differences in telomere dynamics.
- *Sexual size dimorphism* (SSD). Following Freckleton and colleagues [4], the effect of SSD was tested by including male adult body mass (log-transformed) as a covariate in the model while controlling for female adult body mass (log-transformed and added as covariate in the analysis). We chose the SSD because it is a reliable proxy of the sex differences in the energy amount that is allocated into growth and body mass maintenance.
- The *mating system* was included as a three-level factor: polygyny (*i.e.* when males mate with several females during a given reproductive season), monogamy (when both males and females mate with only one partner during a given reproductive season), polyandry (*i.e.* when females mate with several males) and promiscuity (*i.e.* when both sexes mate repeatedly during a given reproductive season). As only few species display strict polyandry in vertebrates, polyandrous and promiscuous species were pooled together.
- *Age at first reproduction* (log-transformed to improve normality) was used as a proxy of the slow-fast continuum of life histories because, in the absence of data on generation time, it is the best proxy of the pace of life [5]. For instance, bird species with fast life histories have a greater telomere attrition than those with slow life histories [6]. Whether this attrition rate differs between sexes and leads to sex differences in adult TL remains unknown.
- The *data quality index* was implemented as a two-level factor “Low quality” for data extracted from a graph *vs.* “High quality” for raw data or when effect sizes were calculated from statistics in the original paper.

Supplementary Tables and figures:


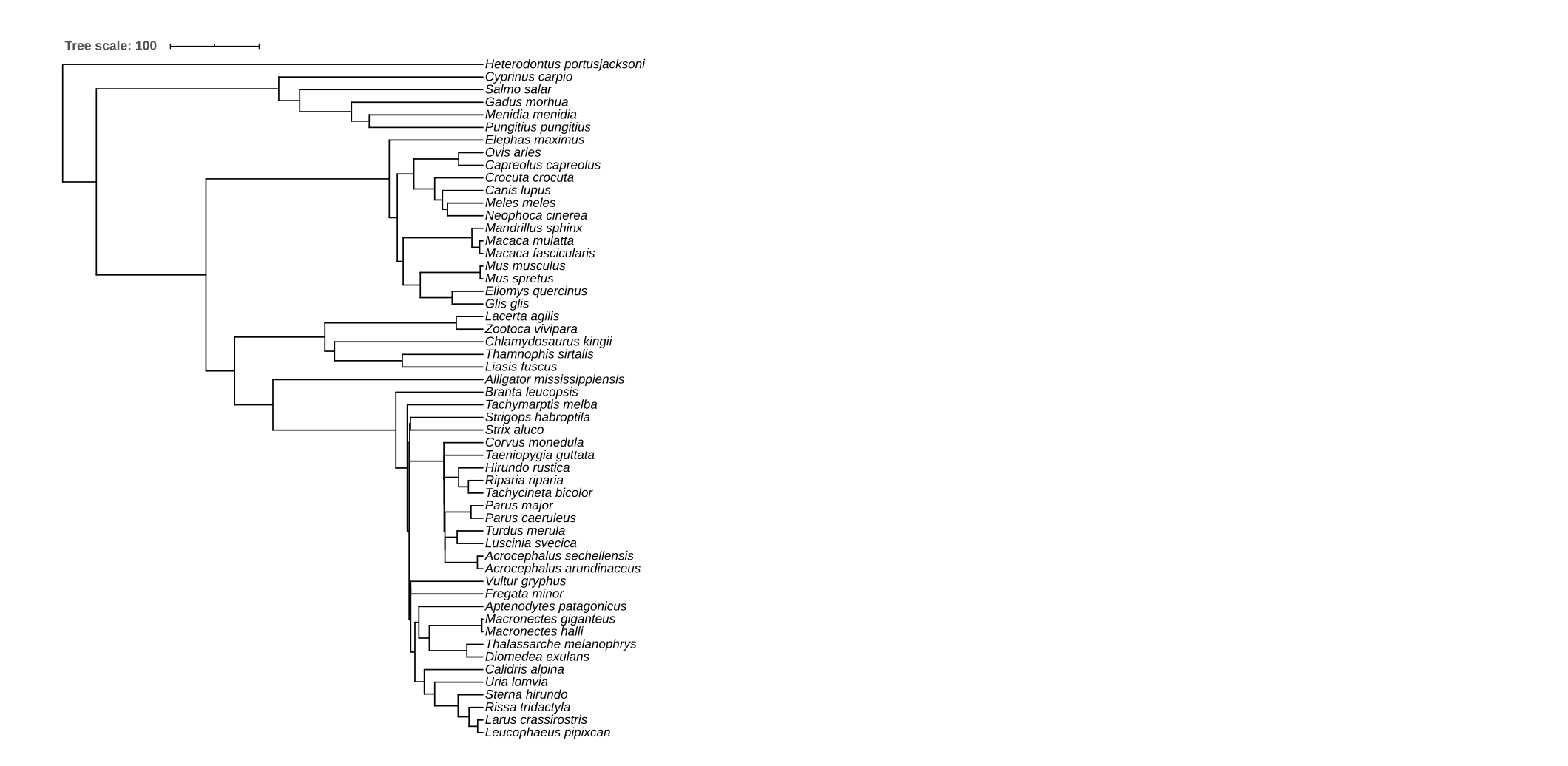


Figure S1: Phylogenetic tree of the 51 species used in the analysis. From <http://www.timetree.org> [10]

Table S1: I² values associated with each random effect (Population, Species and Sample) and the phylogenetic heritability H² value, for the meta-analysis that includes only studies with a sample size strictly higher than 40 individuals. HPDI corresponds to the lower and upper high posterior density limits of the credible interval.

|  | Meta-analysis with sample size > 40 | |
| --- | --- | --- |
|  | Mean | HPDI |
| I² Population | 0.141 | [0.006 : 0.396] |
| I² Species | 0.181 | [0.009 : 0.452] |
| I² Sample | 0.153 | [0.008 : 0.435] |
| I² Residuals | 0.083 | [0.007 : 0.241] |
| H² | 0.336 | [0.014 : 0.693] |
| I² Total | 0.842 | [0.727 : 0.926] |

Table S2: Set of models tested to fit the best age function on telomere length data. Nb corresponds to the number of effect sizes of age-corrected telomere length that were fitted with the corresponding model.

| Models tested | Nb |
| --- | --- |
| Null | 22 |
| Linear | 5 |
| Quadratic | 3 |


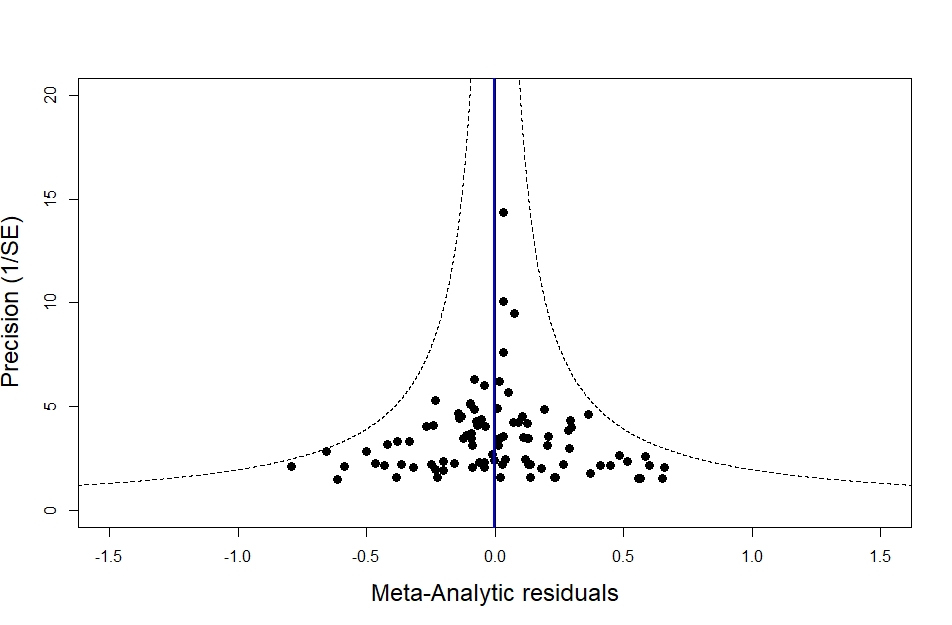

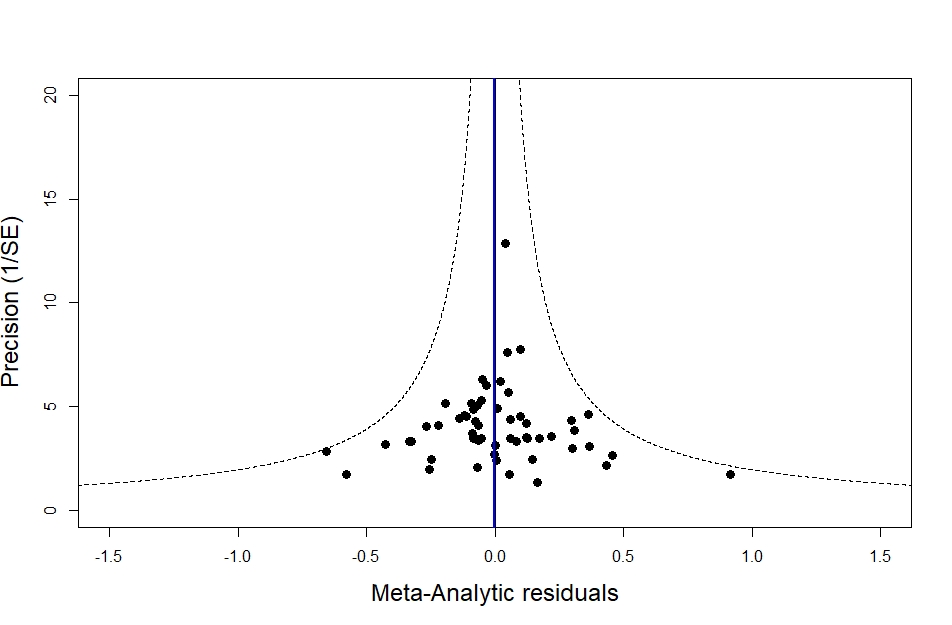

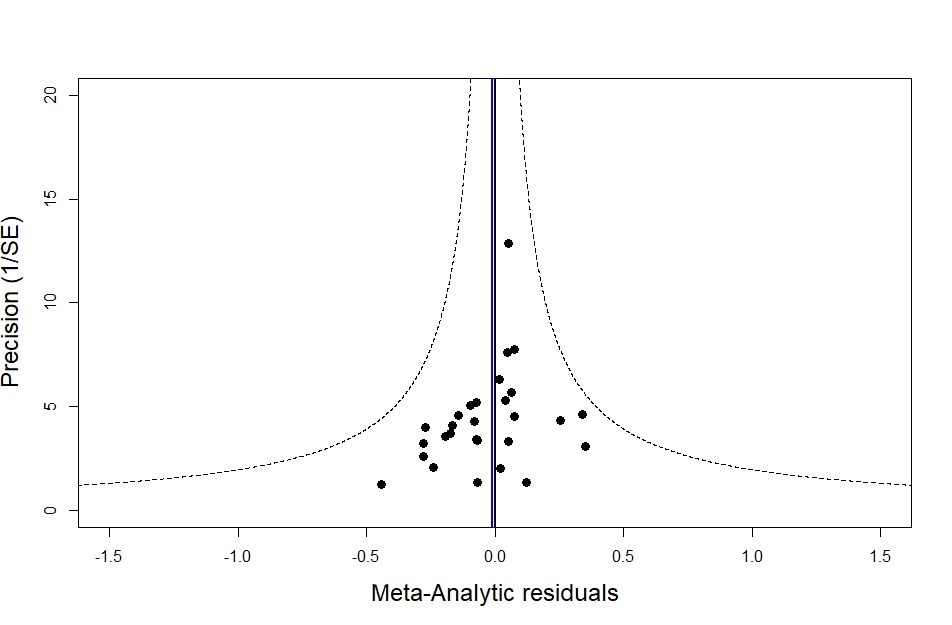


Figure S2: Funnel plots for the first, the second and the third meta-analysis. The precision is plotted against the meta-analytic residuals. The vertical line corresponds to the null meta-analytic residual.


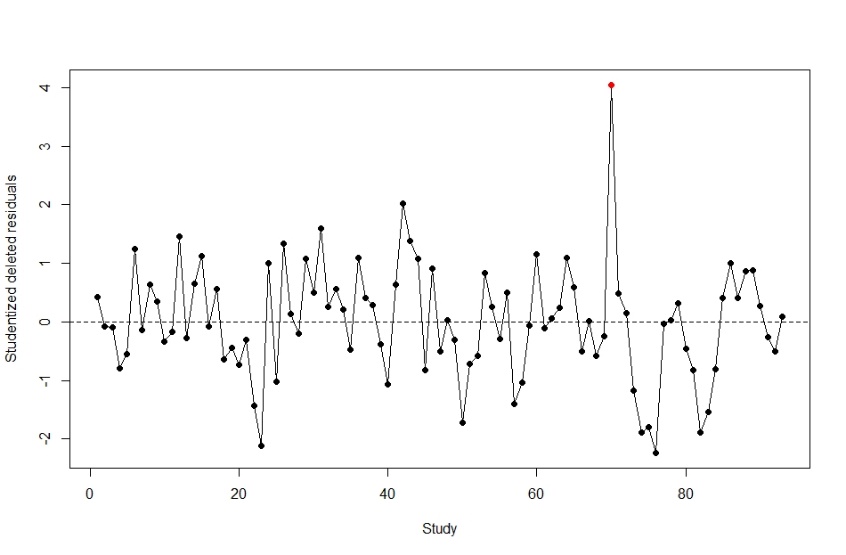
(A)

Studentized deleted residuals

Study


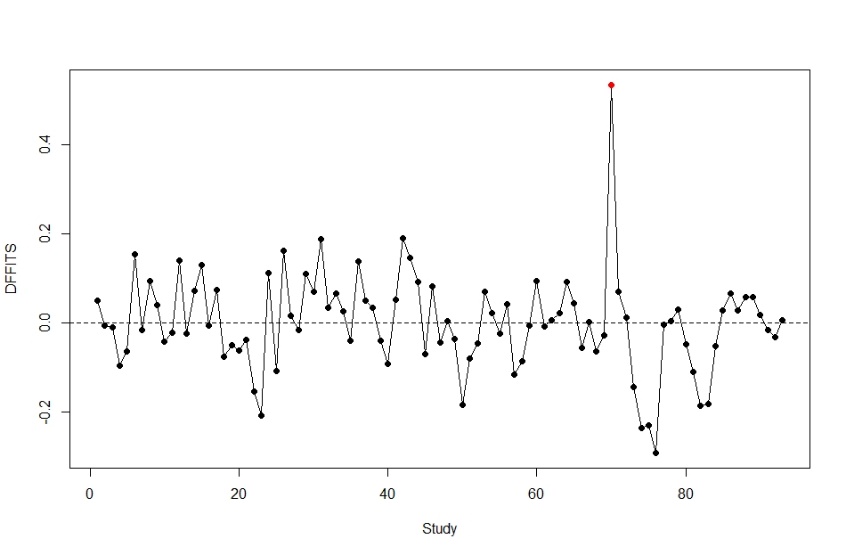
(B)

DFFITS


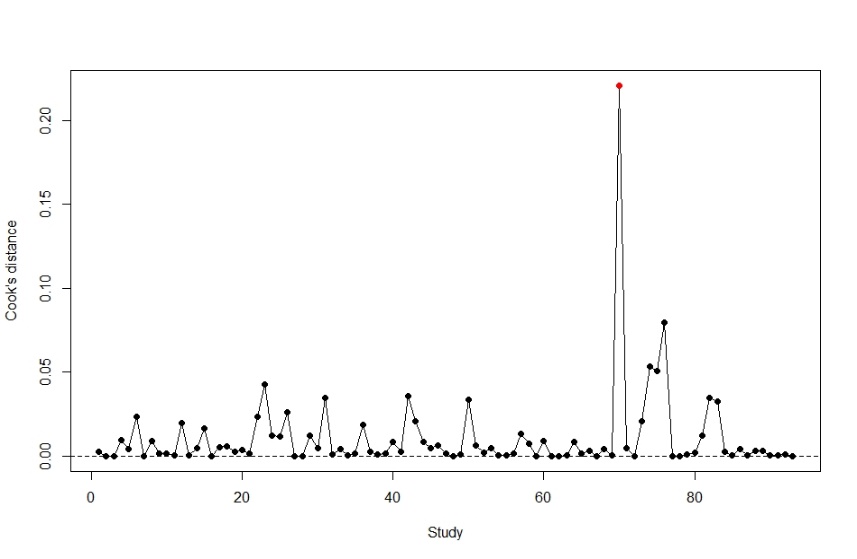
(C)

Cook’s distance

Study

Study

Figure S3: Scatter plots of (A) the studentized deleted residuals, (B) the DFFITS and (C) the Cook’s distance for each case study. The red point represents the study on the Mandrill [9] that we excluded from the analysis.

Table S3: Summary of the number of studies, species and effect size included in the second (adults, non-corrected for age) and the third (age-corrected) meta-analyses.

|  |  | **Meta-analysis 2** | **Meta-analysis 3** |
| --- | --- | --- | --- |
| Number of studies | Total | 46 | 23 |
|  | Mammals | 9 | 8 |
|  | Birds | 27 | 9 |
|  | Reptiles | 5 | 3 |
|  | Fishes | 5 | 3 |
| Number of species | Total | 40 | 22 |
|  | Mammals | 9 | 8 |
|  | Birds | 22 | 9 |
|  | Reptiles | 5 | 3 |
|  | Fishes | 4 | 2 |
| Number of effect sizes | Total | 57 | 30 |
|  | Mammals | 16 | 12 |
|  | Birds | 28 | 10 |
|  | Reptiles | 5 | 3 |
|  | Fishes | 8 | 5 |

**Table S4: *I²* values associated with each random effect (Population, Species and Author) and *H²* value, for the second and the third meta-analyses. HPDI corresponds to the lower and upper high posterior density limits of the credible interval.**

| **Meta-analysis 2** | **Mean** | **HPDI** |
| --- | --- | --- |
| *I²* Population | 0.124 | [0.008 : 0.322] |
| *I²* Species | 0.137 | [0.008 : 0.361] |
| *I²* Sample | 0.144 | [0.008 : 0.366] |
| *I²* Residuals | 0.086 | [0.007 : 0.241] |
| H² | 0.360 | [0.018 : 0.694] |
| *I²* Total | 0.766 | [0.606 : 0.872] |
| **Meta-analysis 3** |  |  |
| *I²* Population | 0.169 | [0.006 : 0.454] |
| *I²* Species | 0.148 | [0.005 : 0.416] |
| *I²* Sample | 0.167 | [0.009 : 0.482] |
| *I²* Residuals | 0.101 | [0.003 : 0.322] |
| H² | 0.311 | [0.012 : 0.679] |
| *I²* Total | 0.848 | [0.703 : 0.944] |

**Table S5: Meta-analysis mean of each moderator with their 95% high posterior density interval (HPDI) for (A) the second (using data on adult only) and (B) the third (using age-corrected data) meta-analyses.**

(A)

|  | **Hedge’s g** | **HPDI** |
| --- | --- | --- |
| Mammal | -0.186 | [-0.730:0.360] |
| Bird | 0.068 | [-0.490:0.549] |
| Reptile | -0.320 | [-0.883:0.310] |
| Fish | -0.005 | [-0.558:0.473] |
| WBC | 0.072 | [-0.440:0.627] |
| RBC | -0.025 | [-0.572:0.454] |
| Other tissues | -0.170 | [-0.610:0.310] |
| TRF _in gel_ | -0.199 | [-0.709:0.254] |
| TRF_southern_ | -0.192 | [-0.629:0.190] |
| qPCR | 0.038 | [-0.303:0.385] |
| Polygynous | -0.113 | [-0.609:0.389] |
| Promiscuous/polyandrous | -0.059 | [-0.463:0.389] |
| Monogamous | -0.077 | [-0.547:0.424] |
| Meta-Analysis mean | -0.064 | [-0.389 :0.263] |

(B)

|  | **Hedge’s g** | **HPDI** |
| --- | --- | --- |
| Mammal | -0.121 | [-0.868:0.711] |
| Bird | 0.093 | [-0.693:0.876] |
| Reptile | -0.409 | [-1.354:0.498] |
| Fish | 0.258 | [-0.485:1.087] |
| WBC | 0.137 | [-0.559:0.922] |
| RBC | -0.021 | [-0.680:0.738] |
| Other tissues | -0.024 | [-0.681:0.614] |
| TRF | -0.211 | [-0.720:0.335] |
| qPCR | 0.112 | [-0.295:0.593] |
| Polygynous | -0.121 | [-0.710:0.429] |
| Promiscuous/polyandrous | 0.070 | [-0.545:0.639] |
| Monogamous | -0.119 | [-0.733:0.529] |
| Meta-Analysis mean | -0.009 | [-0.384:0.410] |


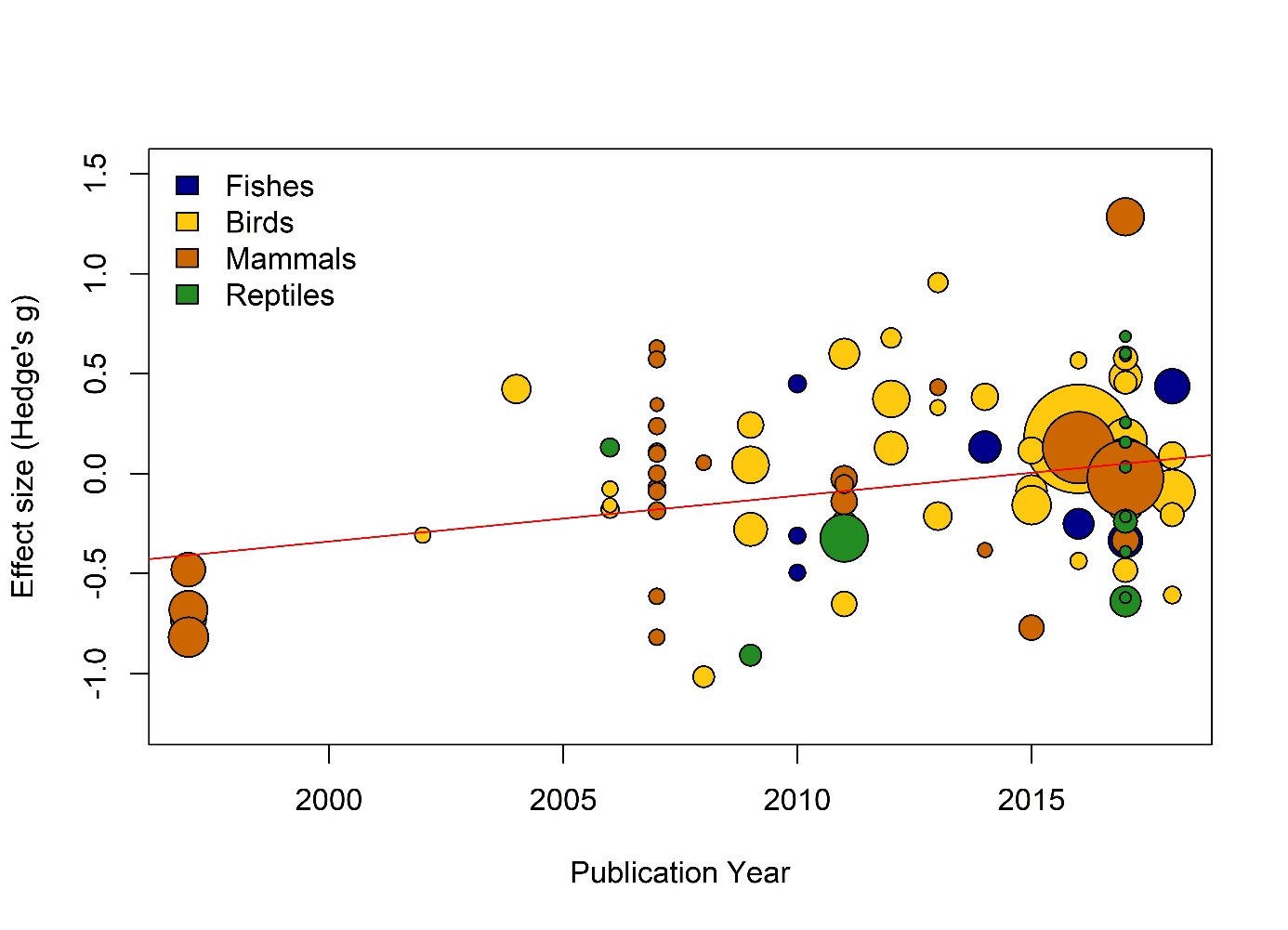


Figure S4: Bubble plot of the effect size (Hedge’s g) across years of publication for the four classes of vertebrates considered in the meta-analyses. Effect sizes are weighted by their precision with larger bubbles indicating more precise estimates and smaller bubbles less precise. The red line represents the slope of the relation, which is not statistically significant

**Table S6: Model selection to test the influence of the sex difference in telomere length (g) on the sex difference in adult life expectancy for (A) the whole dataset, (B) birds only, (C) mammals only. AFR corresponds to Age at First Reproduction, Mass F to mean female adult mass (log-transformed).**

(A)

| Intercept | g | AFR | Mass F | g x AFR | g x Mass F | df | DIC | delta | weight |
| --- | --- | --- | --- | --- | --- | --- | --- | --- | --- |
| -0.384 |  |  | 0.006 |  |  | 5 | -155.893 | 0.000 | 0.258 |
| -0.337 |  |  |  |  |  | 4 | -155.741 | 0.152 | 0.239 |
| -0.439 |  | 0.111 |  |  |  | 5 | -155.672 | 0.221 | 0.231 |
| -0.268 |  | 0.175 | -0.032 |  |  | 6 | -155.508 | 0.385 | 0.213 |
| -0.364 | -0.010 |  | 0.006 |  |  | 6 | -150.279 | 5.614 | 0.016 |
| -0.447 | -0.009 | 0.113 |  |  |  | 6 | -150.129 | 5.764 | 0.014 |
| -0.327 | -0.010 |  |  |  |  | 5 | -150.028 | 5.865 | 0.014 |
| -0.285 | -0.007 | 0.171 | -0.031 |  |  | 7 | -149.816 | 6.077 | 0.012 |
| -0.385 | 0.025 |  | 0.007 |  | -0.008 | 7 | -144.602 | 11.291 | 0.001 |
| -0.277 | 0.022 | 0.176 | -0.031 |  | -0.007 | 8 | -144.544 | 11.348 | 0.001 |
| -0.456 | -0.006 | 0.111 |  | -0.019 |  | 7 | -143.817 | 12.075 | 0.001 |
| -0.267 | -0.007 | 0.171 | -0.030 | -0.017 |  | 8 | -143.435 | 12.458 | 0.001 |
| -0.273 | 0.002 | 0.173 | -0.030 | -0.016 | -0.002 | 9 | -138.479 | 17.414 | 0.000 |

(B)

| Intercept | g | AFR | Mass F | g x AFR | g x Mass F | df | DIC | delta | weight |
| --- | --- | --- | --- | --- | --- | --- | --- | --- | --- |
| -0.072 |  | 0.088 |  |  |  | 5 | -91.509 | 0.000 | 0.252 |
| -0.108 |  |  | 0.017 |  |  | 5 | -91.441 | 0.067 | 0.244 |
| -0.015 |  | 0.115 | -0.014 |  |  | 6 | -91.335 | 0.174 | 0.231 |
| -0.017 |  |  |  |  |  | 4 | -91.246 | 0.263 | 0.221 |
| -0.104 | -0.024 |  | 0.017 |  |  | 6 | -85.892 | 5.617 | 0.015 |
| -0.015 | -0.020 | 0.109 | -0.013 |  |  | 7 | -85.613 | 5.895 | 0.013 |
| -0.068 | -0.022 | 0.088 |  |  |  | 6 | -85.391 | 6.118 | 0.012 |
| -0.009 | -0.024 |  |  |  |  | 5 | -85.155 | 6.354 | 0.011 |
| -0.068 | -0.021 | 0.086 |  | -0.037 |  | 7 | -79.123 | 12.386 | 0.001 |
| -0.014 | 0.058 | 0.105 | -0.010 |  | -0.019 | 8 | -79.059 | 12.449 | 0.000 |
| -0.003 | -0.018 | 0.115 | -0.015 | -0.037 |  | 8 | -78.807 | 12.702 | 0.000 |
| -0.124 | 0.070 |  | 0.021 |  | -0.022 | 7 | -78.338 | 13.171 | 0.000 |
| -0.023 | 0.053 | 0.101 | -0.009 | -0.010 | -0.017 | 9 | -73.660 | 17.849 | 0.000 |

(C)

| Intercept | g | AFR | Mass F | g x AFR | g x Mass F | df | DIC | delta | weight |
| --- | --- | --- | --- | --- | --- | --- | --- | --- | --- |
| -0.511 |  | 0.208 |  |  |  | 5 | -28.740 | 0.000 | 0.160 |
| -0.308 |  |  |  |  |  | 4 | -28.633 | 0.108 | 0.152 |
| 0.600 |  | 0.671 | -0.161 |  |  | 6 | -28.606 | 0.134 | 0.150 |
| -0.262 |  |  | -0.005 |  |  | 5 | -28.596 | 0.144 | 0.149 |
| -0.504 | -0.031 | 0.204 |  |  |  | 6 | -26.882 | 1.858 | 0.063 |
| -0.223 | -0.001 |  | -0.009 |  |  | 6 | -26.638 | 2.102 | 0.056 |
| -0.302 | -0.013 |  |  |  |  | 5 | -26.553 | 2.187 | 0.054 |
| 0.623 | -0.063 | 0.688 | -0.166 |  |  | 7 | -26.358 | 2.382 | 0.049 |
| 0.749 | 0.375 | 0.711 | -0.182 | -0.393 |  | 8 | -26.257 | 2.483 | 0.046 |
| -0.489 | 0.193 | 0.183 |  | -0.185 |  | 7 | -26.124 | 2.617 | 0.043 |
| -0.184 | 0.628 |  | -0.015 |  | -0.070 | 7 | -25.822 | 2.918 | 0.037 |
| 0.747 | 0.780 | 0.692 | -0.183 |  | -0.093 | 8 | -25.501 | 3.239 | 0.032 |
| 0.798 | -0.001 | 0.756 | -0.191 | -0.596 | 0.068 | 9 | -23.121 | 5.619 | 0.010 |

**References for supplementary methods and results**

1. Gardner M *et al.* 2014 Gender and telomere length: systematic review and meta-analysis. *Exp. Gerontol.* **51**, 15–27. (doi:10.1016/j.exger.2013.12.004)

2. Nussey DH *et al.* 2014 Measuring telomere length and telomere dynamics in evolutionary biology and ecology. *Methods in Ecology and Evolution* **5**, 299–310. (doi:10.1111/2041-210X.12161)

3. Tidière M, Gaillard J-M, Berger V, Müller DWH, Bingaman Lackey L, Gimenez O, Clauss M, Lemaître J-F. 2016 Comparative analyses of longevity and senescence reveal variable survival benefits of living in zoos across mammals. *Scientific Reports* **6**, 36361. (doi:10.1038/srep36361)

4. Freckleton RP, Harvey PH, Pagel M, Losos AEJB. 2002 Phylogenetic Analysis and Comparative Data: A Test and Review of Evidence. *The American Naturalist* **160**, 712–726. (doi:10.1086/343873)

5. Gaillard J-M, Yoccoz NG, Lebreton J-D, Bonenfant C, Devillard S, Loison A, Pontier D, Allaine D. 2005 Generation time: a reliable metric to measure life-history variation among mammalian populations. *Am. Nat.* **166**, 119–123. (doi:10.1086/430330)

6. Dantzer B, Fletcher QE. 2015 Telomeres shorten more slowly in slow-aging wild animals than in fast-aging ones. *EXPERIMENTAL GERONTOLOGY* **71**, 38–47. (doi:10.1016/j.exger.2015.08.012)

7. Viechtbauer W, Cheung MW-L. 2010 Outlier and influence diagnostics for meta-analysis. *Research Synthesis Methods* **1**, 112–125. (doi:10.1002/jrsm.11)

8. Viechtbauer W. 2010 Conducting Meta-Analyses in R with the metafor Package. *Journal of Statistical Software* **36**, 1–48. (doi:10.18637/jss.v036.i03)

9. Beaulieu M, Benoit L, Abaga S, Kappeler PM, Charpentier MJE. 2017 Mind the cell: Seasonal variation in telomere length mirrors changes in leucocyte profile. *MOLECULAR ECOLOGY* **26**, 5603–5613. (doi:10.1111/mec.14329)

10. Kumar S, Stecher G, Suleski M, Hedges SB. 2017 TimeTree: A Resource for Timelines, Timetrees, and Divergence Times. *Mol. Biol. Evol.* **34**, 1812–1819. (doi:10.1093/molbev/msx116)

**References used for moderators:**

Ahonen H. 2013 Population structure and mating system of the Australian sea lion (*Neophoca cinerea*). PhD thesis, Université Paris Sud - Paris XI. See <https://tel.archives-ouvertes.fr/tel-01070276/document>.

Amundsen T, Forsgren E, Hansen Lars T. T. 1997 On the function of female ornaments: male bluethroats prefer colourful females. *Proceedings of the Royal Society of London. Series B: Biological Sciences* **264**, 1579–1586. (doi:[10.1098/rspb.1997.0220](https://doi.org/10.1098/rspb.1997.0220))

Andrade MCR, Ribeiro CT, Silva VF da, Molinaro EM, Gonçalves MÂB, Marques MAP, Cabello PH, Leite JPG. 2004 Biologic data of *Macaca mulatta*, *Macaca fascicularis*, and *Saimiri sciureus* used for research at the fiocruz primate center. *Memórias do Instituto Oswaldo Cruz* **99**, 584–589. (doi:[10.1590/S0074-02762004000600009](https://doi.org/10.1590/S0074-02762004000600009))

Barrett ELB, Burke TA, Hammers M, Komdeur J, Richardson DS. 2013 Telomere length and dynamics predict mortality in a wild longitudinal study. *Molecular Ecology* **22**, 249–259. (doi:[10.1111/mec.12110](https://doi.org/10.1111/mec.12110))

Berglind S-Å. 2000 Demography and Management of Relict Sand Lizard *Lacerta agilis* Populations on the Edge of Extinction. *Ecological Bulletins*, 123–142.

Birkhead TR, Burke T, Zann R, Hunter FM, Krupa AP. 1990 Extra-pair paternity and intraspecific brood parasitism in wild zebra finches *Taeniopygia guttata*, revealed by DNA fingerprinting. *Behav Ecol Sociobiol* **27**, 315–324. (doi:[10.1007/BF00164002](https://doi.org/10.1007/BF00164002))

Bize P, Gasparini J, Klopfenstein A, Altwegg R, Roulin A. 2006 Melanin-Based Coloration Is a Nondirectionally Selected Sex-Specific Signal of Offspring Development in the Alpine Swift. *Evolution* **60**, 2370–2380. (doi:[10.1111/j.0014-3820.2006.tb01871.x](https://doi.org/10.1111/j.0014-3820.2006.tb01871.x))

Blomqvist D, Flodin L-Å. 2012 Divorce and breeding dispersal in the dunlin *Calidris alpina*: support for the better option hypothesis? *Behaviour* **149**, 67–80. (doi:[10.1163/156853912X626295](https://doi.org/10.1163/156853912X626295))

Breton AR, Nisbet ICT, Mostello CS, Hatch JJ. 2014 Age-dependent breeding dispersal and adult survival within a metapopulation of Common Terns *Sterna hirundo*. *Ibis* **156**, 534–547. (doi:[10.1111/ibi.12161](https://doi.org/10.1111/ibi.12161))

Bried J, Jiguet F, Jouventin P. 1999 Why Do Aptenodytes Penguins Have High Divorce Rates? *The Auk* **116**, 504–512. (doi:[10.2307/4089382](https://doi.org/10.2307/4089382))

Brom T. 2015 Evolution conjointe des stratégies d’appariement et de dispersion. PhD thesis, Université Pierre et Marie Curie - Paris VI. See <https://tel.archives-ouvertes.fr/tel-01235605/document>.

Brook BW, Griffiths AD. 2004 Frillneck Lizard *Chlamydosaurus kingii* in northern Australia: determining optimal fire management regimes. In *Species Conservation and Management: Case studies*, Oxford University Press.

Brouwer L, Richardson DS, Eikenaar C, Komdeur J. 2006 The role of group size and environmental factors on survival in a cooperatively breeding tropical passerine. *Journal of Animal Ecology* **75**, 1321–1329. (doi:[10.1111/j.1365-2656.2006.01155.x](https://doi.org/10.1111/j.1365-2656.2006.01155.x))

Brown P, Sivakumaran KP, Stoessel D, Giles A. 2005 Population biology of carp (*Cyprinus carpio L.*) in the mid-Murray River and Barmah Forest Wetlands, Australia. *Mar. Freshwater Res.* **56**, 1151–1164. (doi:[10.1071/MF05023](https://doi.org/10.1071/MF05023))

Burger J, Gochfeld M. 1996 Heavy metal and selenium levels in Franklin’s Gull (*Larus pipixcan*) parents and their eggs. *Arch. Environ. Contam. Toxicol.* **30**, 487–491. (doi:[10.1007/BF00213400](https://doi.org/10.1007/BF00213400))

Burger J, Gochfeld M. 1994 Franklin’s Gull (*Larus pipixcan*). *The Birds of North America Online* (doi:[10.2173/bna.116](https://doi.org/10.2173/bna.116))

Campbell R. 2003 Demography and population genetic structure of the Australian sea lion, *Neophoca cinerea*. See <https://research-repository.uwa.edu.au/en/publications/demography-and-population-genetic-structure-of-the-australian-sea>.

Cassaing J, Isaac F. 2007 Pair bonding in the wild mouse *Mus spretus*: inference on the mating system. *Comptes Rendus Biologies* **330**, 828–836. (doi:[10.1016/j.crvi.2007.07.008](https://doi.org/10.1016/j.crvi.2007.07.008))

Chamaillé‐Jammes S, Massot M, Aragón P, Clobert J. 2006 Global warming and positive fitness response in mountain populations of common lizards *Lacerta vivipara*. *Global Change Biology* **12**, 392–402. (doi:[10.1111/j.1365-2486.2005.01088.x](https://doi.org/10.1111/j.1365-2486.2005.01088.x))

Clobert J, Perrins CM, McCleery RH, Gosler AG. 1988 Survival Rate in the Great Tit *Parus major* in Relation to Sex, Age, and Immigration Status. *Journal of Animal Ecology* **57**, 287–306. (doi:[10.2307/4779](https://doi.org/10.2307/4779))

Clutton-Brock TH, Isvaran K. 2007 Sex differences in ageing in natural populations of vertebrates. *Proc Biol Sci* **274**, 3097–3104. (doi:[10.1098/rspb.2007.1138](https://doi.org/10.1098/rspb.2007.1138))

Conover DO, Kynard BE. 1984 Field and laboratory observations of spawning periodicity and behavior of a northern population of the Atlantic silverside, *Menidia menidia* (Pisces: Atherinidae). *Environ Biol Fish* **11**, 161–171. (doi:[10.1007/BF00000462](https://doi.org/10.1007/BF00000462))

Conover DO, Ross MR. 1982 Patterns in seasonal abundance, growth and biomass of the atlantic silverside, *Menidia menidia*, in a New England estuary. *Estuaries* **5**, 275–286. (doi:[10.2307/1351750](https://doi.org/10.2307/1351750))

Conroy JWH. 1972 Ecological aspects of the biology of the Giant Petrel, *Macronectes giganteus* (Gmelin), in the maritime Antarctic - BAS Scientific Report 75.

Cowley E, Siriwardena GM. 2005 Long-term variation in survival rates of Sand Martins *Riparia riparia*: dependence on breeding and wintering ground weather, age and sex, and their population consequences. *Bird Study* **52**, 237–251. (doi:[10.1080/00063650509461397](https://doi.org/10.1080/00063650509461397))

Croxall JP, Rothery P, Pickering SPC, Prince PA. 1990 Reproductive Performance, Recruitment and Survival of Wandering Albatrosses *Diomedea exulans* at Bird Island, South Georgia. *Journal of Animal Ecology* **59**, 775–796. (doi:[10.2307/4895](https://doi.org/10.2307/4895))

Davis LM, Glenn TC, Elsey RM, Dessauer HC, Sawyer RH. 2001 Multiple paternity and mating patterns in the American alligator, *Alligator mississippiensis*. *Molecular Ecology* **10**, 1011–1024. (doi:[10.1046/j.1365-294X.2001.01241.x](https://doi.org/10.1046/j.1365-294X.2001.01241.x))

Dugdale HL, Macdonald DW, Pope LC, Burke T. 2007 Polygynandry, extra-group paternity and multiple-paternity litters in European badger (*Meles meles*) social groups. *Molecular Ecology* **16**, 5294–5306. (doi:[10.1111/j.1365-294X.2007.03571.x](https://doi.org/10.1111/j.1365-294X.2007.03571.x))

Dugdale HL, Pope LC, Newman C, Macdonald DW, Burke T. 2011 Age-specific breeding success in a wild mammalian population: selection, constraint, restraint and senescence. *Molecular Ecology* **20**, 3261–3274. (doi:[10.1111/j.1365-294X.2011.05167.x](https://doi.org/10.1111/j.1365-294X.2011.05167.x))

Dunning JB. 2007 *CRC Handbook of Avian Body Masses, 2nd edition*. See <https://www.crcpress.com/CRC-Handbook-of-Avian-Body-Masses/Jr/p/book/9781420064445>.

Dupont SM, Barbraud C, Chastel O, Delord K, Ruault S, Weimerskirch H, Angelier F. 2018 Young parents produce offspring with short telomeres: A study in a long-lived bird, the Black-browed Albatross (*Thalassarche melanophrys*). *PLOS ONE* **13**, e0193526. (doi:[10.1371/journal.pone.0193526](https://doi.org/10.1371/journal.pone.0193526))

Eason D. 2006 Breeding biology of kakapo (*Strigops habroptilus*) on offshore island sanctuaries, 1990-2002. *Notornis* **53**, 27–36.

Ellegren H. 1991 Stopover Ecology of Autumn Migrating Bluethroats *Luscinia s. svecica* in Relation to Age and Sex. *Ornis Scandinavica (Scandinavian Journal of Ornithology)* **22**, 340–348. (doi:[10.2307/3676506](https://doi.org/10.2307/3676506))

Ernest SKM. 2003 Life history characteristics of placental nonvolant mammals. *Ecology* **84**, 3402–3402. (doi:[10.1890/02-9002](https://doi.org/10.1890/02-9002))

Fay CW, Neves RJ, Pardue GB. 1983 Species Profiles. Life Histories and Environmental Requirements of Coastal Fishes and Invertebrates (Mid-Atlantic). Atlantic silverside. *U.S. Fish and Wildlife Service, Division of Biological Services, FWS/OBS-82/11.10*,27.

Ferretti V, Winkler DW. 2009 Polygyny in the tree swallow *Tachycineta bicolor*: a result of the cost of searching for an unmated male. *Journal of Avian Biology* **40**, 289–295. (doi:[10.1111/j.1600-048X.2008.04519.x](https://doi.org/10.1111/j.1600-048X.2008.04519.x))

Gaillard J-M, Viallefont A, Loison A, Festa-Bianchet M. 2004 Assessing senescence patterns in populations of large mammals. *Animal Biodiversity and Conservation* **27**, 47-58–58.

Gaillard J-M, Sempéré AJ, Boutin J-M, Laere GV, Boisaubert B. 1992 Effects of age and body weight on the proportion of females breeding in a population of roe deer (*Capreolus capreolus*). *Can. J. Zool.* **70**, 1541–1545. (doi:[10.1139/z92-212](https://doi.org/10.1139/z92-212))

Garamszegi LZ, Eens M, Erritzøe J, Møller AP. 2005 Sexually size dimorphic brains and song complexity in passerine birds. *Behav Ecol* **16**, 335–345. (doi:[10.1093/beheco/arh167](https://doi.org/10.1093/beheco/arh167))

Garant D, Dodson JJ, Bernatchez L. 2001 A Genetic Evaluation of Mating System and Determinants of Individual Reproductive Success in Atlantic Salmon (*Salmo salar L*.). *J Hered* **92**, 137–145. (doi:[10.1093/jhered/92.2.137](https://doi.org/10.1093/jhered/92.2.137))

Hammers M, Richardson DS, Burke T, Komdeur J. 2012 Age-Dependent Terminal Declines in Reproductive Output in a Wild Bird. *PLOS ONE* **7**, e40413. (doi:[10.1371/journal.pone.0040413](https://doi.org/10.1371/journal.pone.0040413))

Harcourt AH, Harvey PH, Larson SG, Short RV. 1981 Testis weight, body weight and breeding system in primates. *Nature* **293**, 55. (doi:[10.1038/293055a0](https://doi.org/10.1038/293055a0))

Hasselquist D, Bensch S, Schantz T von. 1995 Low frequency of extrapair paternity in the polygynous great reed warbler, *Acrocephalus arundinaceus*. *Behav Ecol* **6**, 27–38. (doi:[10.1093/beheco/6.1.27](https://doi.org/10.1093/beheco/6.1.27))

Hayward AD, Mar KU, Lahdenperä M, Lummaa V. 2014 Early reproductive investment, senescence and lifetime reproductive success in female Asian elephants. *Journal of Evolutionary Biology* **27**, 772–783. (doi:[10.1111/jeb.12350](https://doi.org/10.1111/jeb.12350))

Hector J a. L, Croxall JP, Follett BK. 1986 Reproductive endocrinology of the Wandering Albatross *Diomedea exulans* in relation to biennial breeding and deferred sexual maturity. *Ibis* **128**, 9–22. (doi:[10.1111/j.1474-919X.1986.tb02088.x](https://doi.org/10.1111/j.1474-919X.1986.tb02088.x))

Henderson IG, Hart PJB, Burke T. 2000 Strict Monogamy in a Semi-Colonial Passerine: The Jackdaw *Corvus monedula*. *Journal of Avian Biology* **31**, 177–182.

Hirons GJM, Hardy AR, Stanley PI. 1984 Body weight, gonad development and moult in the Tawny owl (*Strix alum*). *Journal of Zoology* **202**, 145–164. (doi:[10.1111/j.1469-7998.1984.tb05948.x](https://doi.org/10.1111/j.1469-7998.1984.tb05948.x))

Hirschenhauser K, Winkler H, Oliveira RF. 2003 Comparative analysis of male androgen responsiveness to social environment in birds: the effects of mating system and paternal incubation. *Hormones and Behavior* **43**, 508–519. (doi:[10.1016/S0018-506X(03)00027-8](https://doi.org/10.1016/S0018-506X(03)00027-8))

Holekamp KE, Sakai ST, Lundrigan BL. 2007 Social intelligence in the spotted hyena (*Crocuta crocuta*). *Philos Trans R Soc Lond B Biol Sci* **362**, 523–538. (doi:[10.1098/rstb.2006.1993](https://doi.org/10.1098/rstb.2006.1993))

Hunter S. 1984 Breeding biology and population dynamics of giant petrels *Macronectes* at South Georgia (Aves: Procellariiformes). *Journal of Zoology* **203**, 441–460. (doi:[10.1111/j.1469-7998.1984.tb02343.x](https://doi.org/10.1111/j.1469-7998.1984.tb02343.x))

Hunter S. 1987 Species and sexual isolating mechanisms in sibling species of giant petrels *Macronectes*. *Polar Biol* **7**, 295–301. (doi:[10.1007/BF00443947](https://doi.org/10.1007/BF00443947))

Izzo C. 2010 Patterns of telomere length change with age in aquatic vertebrates and the phylogenetic distribution of the pattern among jawed vertebrates. PhD thesis. University of Adelaide, South Australia.

Izzo C, Rodda KR. 2012 Comparative rates of growth of the Port Jackson shark throughout its southern Australian range. *Mar. Freshwater Res.* **63**, 687–694. (doi:[10.1071/MF11272](https://doi.org/10.1071/MF11272))

Jones OR, Crawley MJ, Pilkington JG, Pemberton JM. 2005 Predictors of early survival in Soay sheep: cohort-, maternal- and individual-level variation. *Proc Biol Sci* **272**, 2619–2625. (doi:[10.1098/rspb.2005.3267](https://doi.org/10.1098/rspb.2005.3267))

Juola F. 2010 Mate Choice in a Sexually Dimorphic Marine Bird, the Great Frigatebird (*Fregata minor*). PhD thesis. University of Miami. See <https://scholarlyrepository.miami.edu/oa_dissertations/500>.

Kazama K, Niizuma Y, Watanuki Y. 2012 Intraspecific Kleptoparasitism, Attacks on Chicks and Chick Adoption in Black-tailed Gulls (*Larus crassirostris*). *Waterbirds: The International Journal of Waterbird Biology* **35**, 599–607.

Khoriauli L *et al.* 2017 Assortative mating for telomere length and antioxidant capacity in barn swallows (*Hirundo rustica*). *Behavioral Ecology and Sociobiology* **71**. (doi:[10.1007/s00265-017-2352-y](https://doi.org/10.1007/s00265-017-2352-y))

Kryštufek B. 2010 *Glis glis* (Rodentia: Gliridae). *Mamm Species* **42**, 195–206. (doi:[10.1644/865.1](https://doi.org/10.1644/865.1))

Kulik L, Amici F, Langos D, Widdig A. 2015 Sex Differences in the Development of Social Relationships in Rhesus Macaques (*Macaca mulatta*). *Int J Primatol* **36**, 353–376. (doi:[10.1007/s10764-015-9826-4](https://doi.org/10.1007/s10764-015-9826-4))

Larsen KW, Gregory PT. 1989 Population Size and Survivorship of the Common Garter Snake, *Thamnophis sirtalis*, near the Northern Limit of Its Distribution. *Holarctic Ecology* **12**, 81–86.

Latham N, Mason G. 2004 From house mouse to mouse house: the behavioural biology of free-living *Mus musculus* and its implications in the laboratory. *Applied Animal Behaviour Science* **86**, 261–289. (doi:[10.1016/j.applanim.2004.02.006](https://doi.org/10.1016/j.applanim.2004.02.006))

Leconte M, Paucot C, Dupuy F, Couzi L, Cardonnel S, Bulens P. 2012 Étude de la population de Gorgebleue à miroir blanc *(Luscinia svecica namnetum Mayaud*, 1934 ; Muscicapidae) se reproduisant autour du Bassin d’Arcachon (France, Gironde, 33). *Bull. Soc. Linn. Bordeaux* **147**, 15.

Ludwigs J-D, Becker PH. 2004 What do pairing patterns in common tern, *Sterna hirundo*, recruits reveal about the significance of sex and breeding experience? *Behavioral Ecology and Sociobiology* **57**, 412–421. (doi:[10.1007/s00265-004-0880-8](https://doi.org/10.1007/s00265-004-0880-8))

Macleod R, Barnett P, Clark JA, Cresswell W. 2005 Body mass change strategies in blackbirds *Turdus merula*: the starvation–predation risk trade-off. *Journal of Animal Ecology* **74**, 292–302. (doi:[10.1111/j.1365-2656.2005.00923.x](https://doi.org/10.1111/j.1365-2656.2005.00923.x))

Madsen T, Ujvari B, Shine R, Olsson M. 2006 Rain, rats and pythons: Climate-driven population dynamics of predators and prey in tropical Australia. *Austral Ecology* **31**, 30–37. (doi:[10.1111/j.1442-9993.2006.01540.x](https://doi.org/10.1111/j.1442-9993.2006.01540.x))

Mar KU. 2002 The demography and life history strategies of timber elephants in Myanmar. PhD thesis, University of London. See <http://discovery.ucl.ac.uk/1446021/>.

Massot M, Clobert J, Montes‐Poloni L, Haussy C, Cubo J, Meylan S. 2011 An integrative study of ageing in a wild population of common lizards. *Functional Ecology* **25**, 848–858. (doi:[10.1111/j.1365-2435.2011.01837.x](https://doi.org/10.1111/j.1365-2435.2011.01837.x))

Mclennan D, Armstrong JD, Stewart DC, Mckelvey S, Boner W, Monaghan P, Metcalfe NB. 2016 Interactions between parental traits, environmental harshness and growth rate in determining telomere length in wild juvenile salmon. *Molecular Ecology* **25**, 5425–5438. (doi:[10.1111/mec.13857](https://doi.org/10.1111/mec.13857))

Meikle DB, Vessey SH. 1988 Maternal dominance rank and lifetime survivorship of male and female rhesus monkeys. *Behav Ecol Sociobiol* **22**, 379–383. (doi:[10.1007/BF00294974](https://doi.org/10.1007/BF00294974))

Møller AP. 2006 Sociality, age at first reproduction and senescence: comparative analyses of birds. *Journal of Evolutionary Biology* **19**, 682–689. (doi:[10.1111/j.1420-9101.2005.01065.x](https://doi.org/10.1111/j.1420-9101.2005.01065.x))

Moller AP, Birkhead TR. 1993 Cuckoldry and sociality: a comparative study of birds. *Am. Nat.* **142**, 118–140. (doi:[10.1086/285531](https://doi.org/10.1086/285531))

Møller AP. 1995 Sexual selection in the barn swallow (*Hirundo rustica*). V. Geographic variation in ornament size. *Journal of Evolutionary Biology* **8**, 3–19. (doi:[10.1046/j.1420-9101.1995.8010003.x](https://doi.org/10.1046/j.1420-9101.1995.8010003.x))

Møller AP, Szép T. 2002 Survival Rate of Adult Barn Swallows *Hirundo Rustica* in Relation to Sexual Selection and Reproduction. *Ecology* **83**, 2220–2228. (doi:[10.1890/0012-9658(2002)083[2220:SROABS]2.0.CO;2](https://doi.org/10.1890/0012-9658(2002)083%5b2220:SROABS%5d2.0.CO;2))

Morrow EH, Fricke C. 2004 Sexual selection and the risk of extinction in mammals. *Proceedings of the Royal Society of London. Series B: Biological Sciences* **271**, 2395–2401. (doi:[10.1098/rspb.2004.2888](https://doi.org/10.1098/rspb.2004.2888))

Mumby HS, Chapman SN, Crawley JAH, Mar KU, Htut W, Thura Soe A, Aung HH, Lummaa V. 2015 Distinguishing between determinate and indeterminate growth in a long-lived mammal. *BMC Evolutionary Biology* **15**, 214. (doi:[10.1186/s12862-015-0487-x](https://doi.org/10.1186/s12862-015-0487-x))

Newton I, McGrady MJ, Oli MK. 2016 A review of survival estimates for raptors and owls. *Ibis* **158**, 227–248. (doi:[10.1111/ibi.12355](https://doi.org/10.1111/ibi.12355))

Nichols JD. 1976 Simulation of a commercially harvested alligator population in Louisiana. 61.

Okuda N, Tayasu I, Yanagisawa Y. 1998 Determinate growth in a paternal mouthbrooding fish whose reproductive success is limited by buccal capacity. *Evolutionary Ecology* **12**, 681–699. (doi:[10.1023/A:1006533531952](https://doi.org/10.1023/A:1006533531952))

Olsson M, Madsen T. 2001 Promiscuity in Sand Lizards (*Lacerta agilis*) and Adder Snakes (*Vipera berus*): Causes and Consequences. *J Hered* **92**, 190–197. (doi:[10.1093/jhered/92.2.190](https://doi.org/10.1093/jhered/92.2.190))

Olsson M. 1988 Ecology of a Swedish population of the sand lizard (*Lacerta agilis*) - a preliminary report. *Mertensiella*

Owen M, Black JM. 1989 Factors Affecting the Survival of Barnacle Geese on Migration from the Breeding Grounds. *Journal of Animal Ecology* **58**, 603–617. (doi:[10.2307/4851](https://doi.org/10.2307/4851))

Owens IPF, Hartley IR. 1998 Sexual dimorphism in birds: why are there so many different forms of dimorphism? *Proceedings of the Royal Society of London. Series B: Biological Sciences* **265**, 397–407. (doi:[10.1098/rspb.1998.0308](https://doi.org/10.1098/rspb.1998.0308))

Owens IPF, Bennett Peter M. 1994 Mortality costs of parental care and sexual dimorphism in birds. *Proceedings of the Royal Society of London. Series B: Biological Sciences* **257**, 1–8. (doi:[10.1098/rspb.1994.0086](https://doi.org/10.1098/rspb.1994.0086))

Palomo LJ, Justo ER, Vargas JM. 2009 *Mus spretus* (Rodentia: Muridae). *Mamm Species* , 1–10. (doi:[10.1644/840.1](https://doi.org/10.1644/840.1))

Pauliny A, Larsson K, Blomqvist D. 2012 Telomere dynamics in a long-lived bird, the barnacle goose. *BMC Evolutionary Biology* **12**, 257. (doi:[10.1186/1471-2148-12-257](https://doi.org/10.1186/1471-2148-12-257))

Paz-y-Miño C, Navarrete J, Sánchez ME, Gaviria A, Leone PE, Cabrera-Andrade A, López-Cortés A, Burgos G. 2015 Development of a multiplex system for identifying individuals of Andean Condor (*Vultur gryphus*). *Forensic Science International: Genetics Supplement Series* **5**, e228–e230. (doi:[10.1016/j.fsigss.2015.09.091](https://doi.org/10.1016/j.fsigss.2015.09.091))

Preston BT, Stevenson IR, Lincoln GA, Monfort SL, Pilkington JG, Wilson K. 2012 Testes size, testosterone production and reproductive behaviour in a natural mammalian mating system. *Journal of Animal Ecology* **81**, 296–305. (doi:[10.1111/j.1365-2656.2011.01907.x](https://doi.org/10.1111/j.1365-2656.2011.01907.x))

Prince PA, Rothery P, Croxall JP, Wood AG. 1994 Population dynamics of Black-browed and Grey-headed Albatrosses *Diomedea melanophris* and *D. chrysostoma* at Bird Island, South Georgia. *Ibis* **136**, 50–71. (doi:[10.1111/j.1474-919X.1994.tb08131.x](https://doi.org/10.1111/j.1474-919X.1994.tb08131.x))

Robinson RA, Kew JJ, Kew AJ. 2010 Survival of suburban blackbirds *Turdus merula* varies seasonally but not by sex. *Journal of Avian Biology* **41**, 83–87. (doi:[10.1111/j.1600-048X.2009.04789.x](https://doi.org/10.1111/j.1600-048X.2009.04789.x))

Rollings N, Uhrig EJ, Krohmer RW, Waye HL, Mason RT, Olsson M, Whittington CM, Friesen CR. 2017 Age-related sex differences in body condition and telomere dynamics of red-sided garter snakes. *Proceedings of the Royal Society B: Biological Sciences* **284**. (doi:[10.1098/rspb.2016.2146](https://doi.org/10.1098/rspb.2016.2146))

Ruf T, Fietz J, Schlund W, Bieber C. 2006 High Survival in Poor Years: Life History Tactics Adapted to Mast Seeding in the Edible Dormouse. *Ecology* **87**, 372–381. (doi:[10.1890/05-0672](https://doi.org/10.1890/05-0672))

Sæther B-E, Engen S, Lande R, Møller AP, Bensch S, Hasselquist D, Beier J, Leisler B. 2004 Time to extinction in relation to mating system and type of density regulation in populations with two sexes. *Journal of Animal Ecology* **73**, 925–934. (doi:[10.1111/j.0021-8790.2004.00869.x](https://doi.org/10.1111/j.0021-8790.2004.00869.x))

Saladin V, Ritschard M, Roulin A, Bize P, Richner H. 2007 Analysis of genetic parentage in the tawny owl (*Strix aluco*) reveals extra-pair paternity is low. *J Ornithol* **148**, 113–116. (doi:[10.1007/s10336-006-0109-x](https://doi.org/10.1007/s10336-006-0109-x))

Schaub M, Vaterlaus‐Schlegel C. 2001 Annual and seasonal variation of survival rates in the garden dormouse (*Eliomys quercinus*). *Journal of Zoology* **255**, 89–96. (doi:[10.1017/S0952836901001133](https://doi.org/10.1017/S0952836901001133))

Setchell JM. 2016 Sexual Selection and the differences between the sexes in Mandrills (*Mandrillus sphinx*). *American Journal of Physical Anthropology* **159**, 105–129. (doi:[10.1002/ajpa.22904](https://doi.org/10.1002/ajpa.22904))

Shaffer SA, Weimerskirch H, Costa DP. 2001 Functional significance of sexual dimorphism in Wandering Albatrosses, *Diomedea exulans*. *Functional Ecology* **15**, 203–210. (doi:[10.1046/j.1365-2435.2001.00514.x](https://doi.org/10.1046/j.1365-2435.2001.00514.x))

Shutler D, Clark RG. 2003 Causes and Consequences of Tree Swallow (*Tachycineta Bicolor*) Dispersal in Saskatchewan. *Auk* **120**, 619–631. (doi:[10.1642/0004-8038(2003)120[0619:CACOTS]2.0.CO;2](https://doi.org/10.1642/0004-8038(2003)120%5b0619:CACOTS%5d2.0.CO;2))

Smith PA, Gaston AJ. 2012 Environmental variation and the demography and diet of thick-billed murres. *Marine Ecology Progress Series* **454**, 237–249. (doi:[10.3354/meps09589](https://doi.org/10.3354/meps09589))

Soikkeli M. 1967 Breeding cycle and population dynamics in the dunlin (*Calidris alpina*). *Annales Zoologici Fennici* **4**, 158–198.

Speziale KL, Lambertucci SA, Olsson O. 2008 Disturbance from roads negatively affects Andean condor habitat use. *Biological Conservation* **141**, 1765–1772. (doi:[10.1016/j.biocon.2008.04.017](https://doi.org/10.1016/j.biocon.2008.04.017))

Stockley P, Gage MJ, Parker GA, Møller AP. 1997 Sperm competition in fishes: the evolution of testis size and ejaculate characteristics. *Am. Nat.* **149**, 933–954. (doi:[10.1086/286031](https://doi.org/10.1086/286031))

Sukumar R. 2006 A brief review of the status, distribution and biology of wild Asian elephants *Elephas maximus*. *International Zoo Yearbook* **40**, 1–8. (doi:[10.1111/j.1748-1090.2006.00001.x](https://doi.org/10.1111/j.1748-1090.2006.00001.x))

Svensson E, Nilsson J-A. 1995 Food Supply, Territory Quality, and Reproductive Timing in the Blue Tit (*Parus Caeruleus*). *Ecology* **76**, 1804–1812. (doi:[10.2307/1940712](https://doi.org/10.2307/1940712))

Temrin H, Tullberg BS. 1995 A phylogenetic analysis of the evolution of avian mating systems in relation to altricial and precocial young. *Behav Ecol* **6**, 296–307. (doi:[10.1093/beheco/6.3.296](https://doi.org/10.1093/beheco/6.3.296))

Thomas GH, Székely T, Reynolds JD. 2007 Sexual Conflict and the Evolution of Breeding Systems in Shorebirds. In *Advances in the Study of Behavior*, pp. 279–342. Academic Press. (doi:[10.1016/S0065-3454(07)37006-X](https://doi.org/10.1016/S0065-3454(07)37006-X))

Tickell WLN. 2013 The Biology of the Great Albatrosses, *Diomedea Exulans* and *Diomedea Epomophora*. In *Antarctic Bird Studies*, pp. 1–56. American Geophysical Union (AGU). (doi:[10.1029/AR012p0001](https://doi.org/10.1029/AR012p0001))

Toïgo C, Gaillard J-M. 2003 Causes of sex-biased adult survival in ungulates: sexual size dimorphism, mating tactic or environment harshness? *Oikos* **101**, 376–384. (doi:[10.1034/j.1600-0706.2003.12073.x](https://doi.org/10.1034/j.1600-0706.2003.12073.x))

Tovar-Ávila J, Walker TI, Day RW. 2007 Reproduction of *Heterodontus portusjacksoni* in Victoria, Australia: evidence of two populations and reproductive parameters for the eastern population. *Mar. Freshwater Res.* **58**, 956–965. (doi:[10.1071/MF06230](https://doi.org/10.1071/MF06230))

Ujvari B, Biro PA, Charters JE, Brown G, Heasman K, Beckmann C, Madsen T. 2017 Curvilinear telomere length dynamics in a squamate reptile. *Functional Ecology* **31**, 753–759. (doi:[10.1111/1365-2435.12764](https://doi.org/10.1111/1365-2435.12764))

Valle CA, Vries DE, Hernandez T& C. 2006 Plumage and sexual maturation in the Great Frigatebird *Fregata minor* in the Galapagos Islands. *Marine Ornithology* **34**, 51–59.

Vanpé C, Gaillard J-M, Kjellander P, Liberg O, Delorme D, Hewison AJM. 2010 Assessing the intensity of sexual selection on male body mass and antler length in roe deer Capreolus capreolus: is bigger better in a weakly dimorphic species? *Oikos* **119**, 1484–1492. (doi:[10.1111/j.1600-0706.2010.18312.x](https://doi.org/10.1111/j.1600-0706.2010.18312.x))

Vitt LJ, Caldwell JP. 2013 *Herpetology: An Introductory Biology of Amphibians and Reptiles*. Academic Press.

Watson RL *et al.* 2017 Sex differences in leucocyte telomere length in a free-living mammal. *Molecular Ecology* **26**, 3230–3240. (doi:[10.1111/mec.13992](https://doi.org/10.1111/mec.13992))

Weimerskirch H, Stahl JC, Jouventin P. 1992 The breeding biology and population dynamics of King Penguins *Aptenodytes patagonica* on the Crozet Islands. *Ibis* **134**, 107–117. (doi:[10.1111/j.1474-919X.1992.tb08387.x](https://doi.org/10.1111/j.1474-919X.1992.tb08387.x))

Whittier JM, Mason RT, Crews D. 1985 Mating in the red-sided garter snake, *Thamnophis sirtalis parietalis*: differential effects on male and female sexual behavior. *Behav Ecol Sociobiol* **16**, 257–261. (doi:[10.1007/BF00310989](https://doi.org/10.1007/BF00310989))

Wiese FK, Robertson GJ, Gaston AJ. 2004 Impacts of chronic marine oil pollution and the murre hunt in Newfoundland on thick-billed murre *Uria lomvia* populations in the eastern Canadian Arctic. *Biological Conservation* **116**, 205–216. (doi:[10.1016/S0006-3207(03)00191-5](https://doi.org/10.1016/S0006-3207(03)00191-5))

Wilbourn RV. *et al.* 2017 Age-dependent associations between telomere length and environmental conditions in roe deer. *Biology Letters* **13**, 20170434. (doi:[10.1098/rsbl.2017.0434](https://doi.org/10.1098/rsbl.2017.0434))

Wilkinson PM, Rainwater TR, Woodward AR, Leone EH, Carter C. 2016 Determinate Growth and Reproductive Lifespan in the American Alligator (*Alligator mississippiensis*): Evidence from Long-term Recaptures. *cope* **104**, 843–853. (doi:[10.1643/CH-16-430](https://doi.org/10.1643/CH-16-430))

Wooller RD, Coulson JC. 1977 Factors Affecting the Age of First Breeding of the Kittiwake *Rissa Tridactyla*. *Ibis* **119**, 339–349. (doi:[10.1111/j.1474-919X.1977.tb08252.x](https://doi.org/10.1111/j.1474-919X.1977.tb08252.x))

Xie L *et al.* 2013 Age- and Sex-Based Hematological and Biochemical Parameters for *Macaca fascicularis*. *PLOS ONE* **8**, e64892. (doi:[10.1371/journal.pone.0064892](https://doi.org/10.1371/journal.pone.0064892))

Zann R, Runciman D. 1994 Survivorship, dispersal and sex ratios of Zebra Finches *Taeniopygia guttata* in southeast Australia. *Ibis* **136**, 136–143. (doi:[10.1111/j.1474-919X.1994.tb01077.x](https://doi.org/10.1111/j.1474-919X.1994.tb01077.x))
