# Supplementary figures and images for: No sex differences in adult telomere length across vertebrates: a meta-analysis
Source: R Soc Open Sci. 2020 Nov 11;7(11):200548. doi: 10.1098/rsos.200548 (PMC7735339; doi:10.1098/rsos.200548)

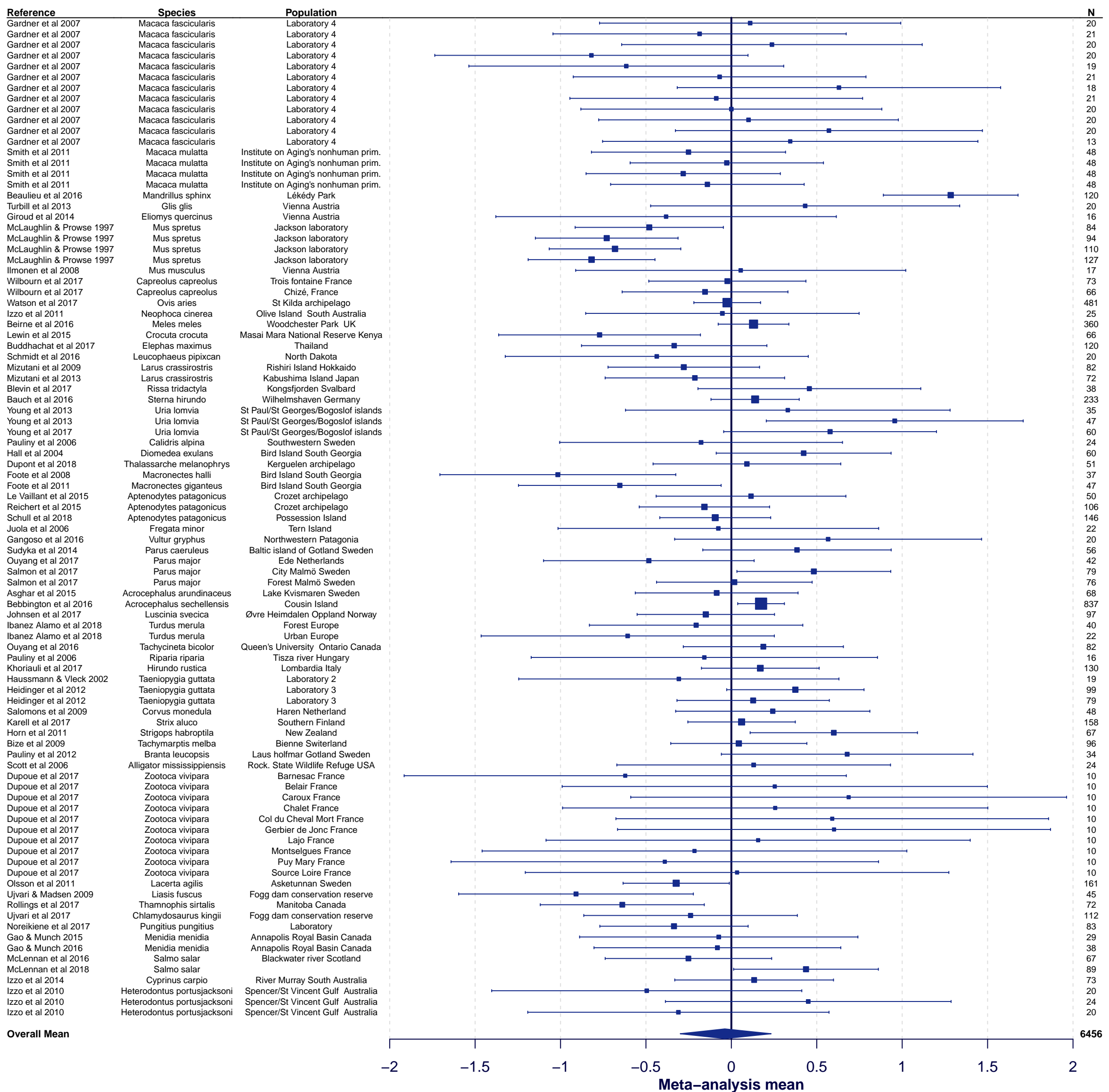

Supplement: Forest plot for the first meta-analysis (juveniles and adult) [file rsos200548supp4.pdf]

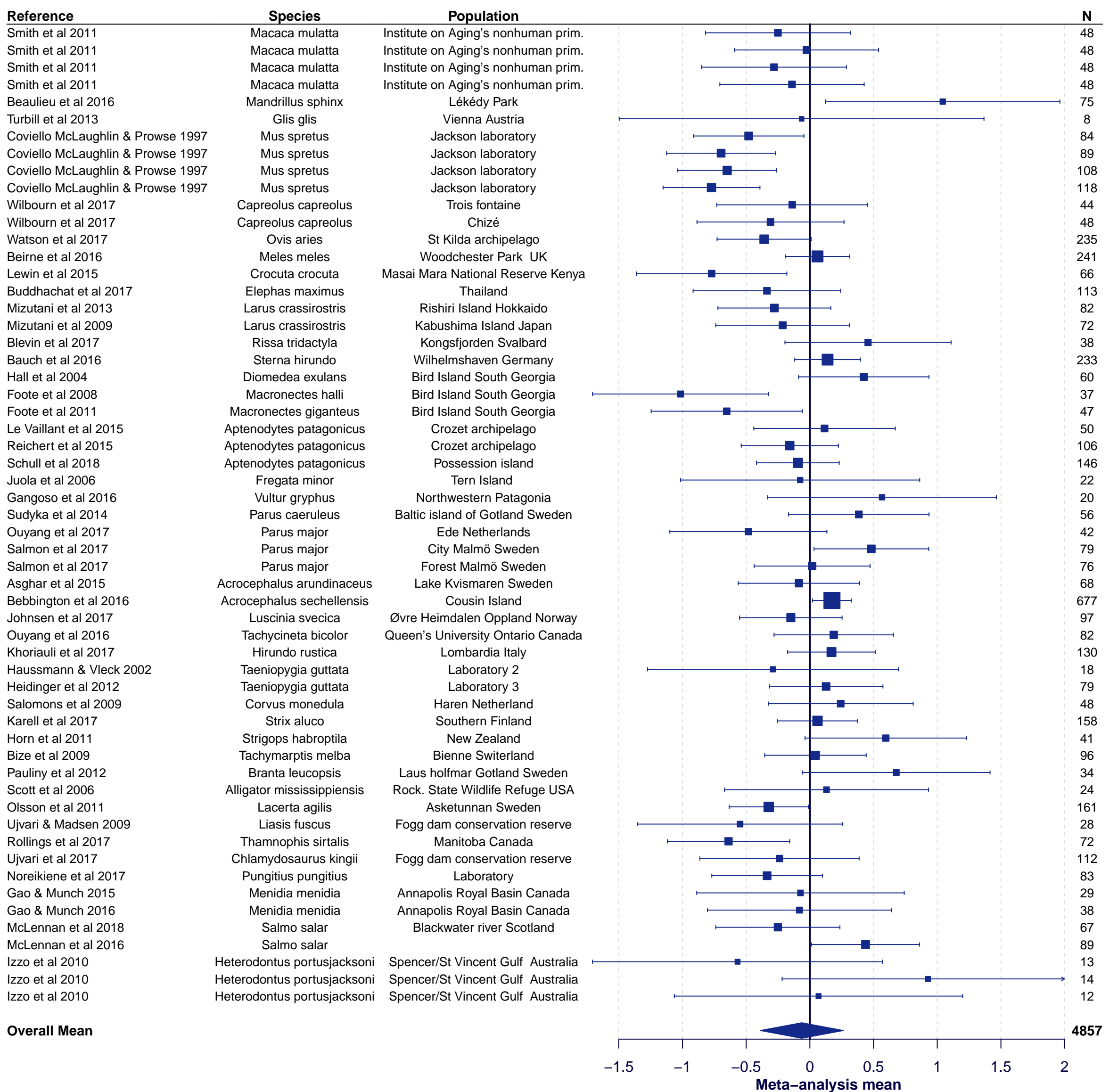

Supplement: Forest plot for the second meta-analysis (adult only) [file rsos200548supp5.pdf]

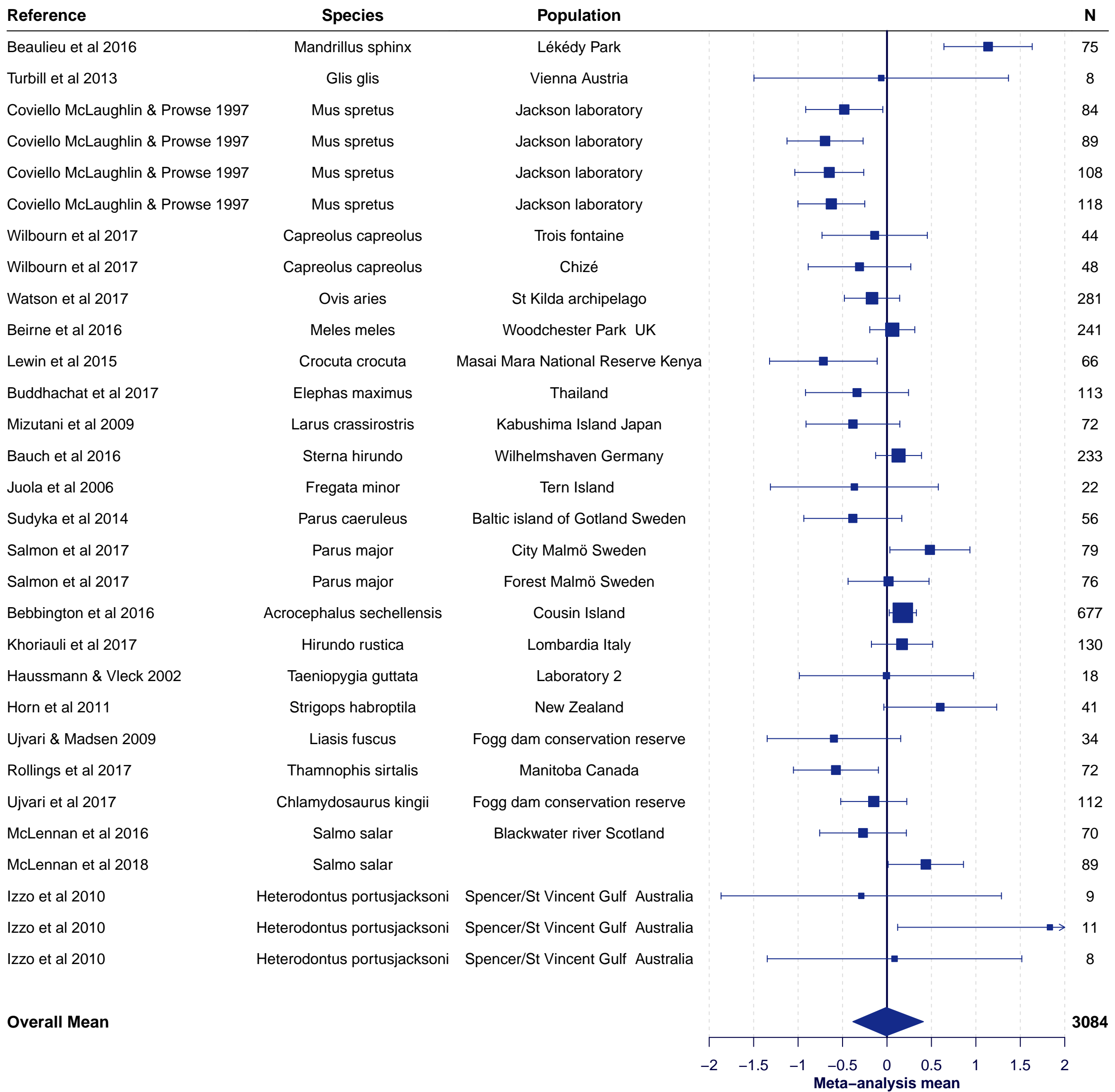

Supplement: Forest plot for the third meta-analysis (adult only age-corrected) [file rsos200548supp6.pdf]
